# *SQUAMOSA* Promoter Binding Protein-Like (*SPL*) Gene Family: TRANSCRIPTOME-Wide Identification, Phylogenetic Relationship, Expression Patterns and Network Interaction Analysis in *Panax ginseng* C. A. Meyer

**DOI:** 10.3390/plants9030354

**Published:** 2020-03-11

**Authors:** Shaokun Li, Li Li, Yang Jiang, Jun Wu, Honghua Sun, Mingzhu Zhao, Yue Jiang, Lei Zhu, Yanfang Wang, Yingjie Su, Kangyu Wang, Yi Wang, Meiping Zhang

**Affiliations:** 1 College of Life Science, Jilin Agricultural University, Changchun 130118, China; lishaokun1168@outlook.com (S.L.); lili910607@sina.com (L.L.); wky427@sina.com (Y.J.); 1398608555@sina.com (J.W.); S15567768463@126.com (H.S.); zhaomingzhu0125@aliyun.com (M.Z.); jiangyue92815@sina.com (Y.J.); zhulei916@sina.com (L.Z.); suyj0923@sina.com (Y.S.); 2 Research Center Ginseng Genetic Resources Development and Utilization, Changchun 130118, China; 3 College of Chinese Medicinal Materials, Jilin Agricultural University, Changchun 130118, China; yfwang2014@sina.com

**Keywords:** *Panax ginseng* C. A. Meyer, SPL transcription factor, Phylogenetic relationship, Gene expression patterns, Network interaction

## Abstract

*SPL* (*SQUAMOSA* promoter binding protein-like) gene family is specific transcription factor in the plant that have an important function for plant growth and development. Although the *SPL* gene family has been widely studied and reported in many various plant species from gymnosperm to angiosperm, there are no systematic studies and reports about the *SPL* gene family in *Panax ginseng* C. A. Meyer. In this study, we conducted transcriptome-wide identification, evolutionary analysis, structure analysis, and expression characteristics analysis of *SPL* gene family in *Panax ginseng* by bioinformatics. We annotated the *PgSPL* gene family and found that they might involve in multiple functions including encoding structural proteins, but the main function were still focused on the binding function. The result showed that 106 *PgSPL* transcripts were classified into two clades - A and B, both of which respectively consisted of three groups. Besides, we profiled *PgSPL* transcripts’ genotypic, temporal, and spatial expression characteristics. Furthermore, we calculated the correlation of *PgSPL* transcripts in the 14 tissues of a 4 years old ginseng and 42 farmers’ cultivars farmers’ cultivars of 4 years old ginsengs’ roots with both results showing that *SPL* transcripts formed a single network, which indicated that *PgSPL*s inter-coordinated when performing their functions. What’s more, we found that most *PgSPL* transcripts tended to express in older ginseng instead of younger ginseng, which was not only reflected in the expression of more types of *SPL* transcripts in older ginseng, but also in the higher expression of *SPL* transcripts in older ginseng. Additionally, we found that four *PgSPL* transcripts were only massively expressed in roots. According to *PgSPL* transcripts’ expression characteristics, we found that *PgSPL23-35* and *PgSPL24-09* were most proper two transcripts to further study as ginseng age’s molecular marker. These results provide the basis for further elucidation of the *PgSPL* transcripts’ biological function in ginseng and ginseng genetics improvement and gene breeding in the future.

## 1. Introduction

Transcription factors (TFs) are a group of protein that can specifically bind to the sequence upstream of a gene to regulate the spatial or temporal expression of it [1]. *SQUAMOSA promoter binding protein-like* (*SPL*) gene family is a kind of TFs that encode the *SQUAMOSA* promoter binding proteins (SBPs). It was first identified in *Antirrhinum majus* with the function of regulating *MADS-box* genes in the early stage of flower development [2]. In the next two decades, the SPL gene family was identified and widely reported in various species such as moss [3], Arabidopsis [4], rice [5], tomato [6], and maize [7]. Through the findings of these study, the *SPL* gene family has multiple biological functions in plant growth and development including flowering time [8,9], sporocytes formation [10] and leaf initiation [11,12]. In particular, the *SPL* genes act as the target of miR156 that controlling the phase transitions between vegetative and reproductive, which is critical to plant reproductive success [13]. Additionally, *SPL* transcripts are also involved in copper homeostasis, fertility and response to GA (gibberellin acids) signaling [14,15].

The *SPL* proteins share the SBP domain, which is consisting of 76 amino acid residues that encoded two zinc fingers and a nuclear localization sequence (NLS). It has the function of binding to the GTAC core sequence and allow it to come into play [16]. In Arabidopsis, the *SPL* gene family can be divided into two categories based on the gene sequence and structure. The members of the second category, except *SPL*8, contain miR156/157 binding sites [14]. The miR156/157 bind to *SPL* transcript and regulate its expression by transcript cleavage or translational repression [17]. Besides, they are important mediators linking age to plant growth and development [18]. The miR156/157 is currently the only one known as an age-based molecular marker [19]. As the target genes of miR156/157, the *SPL* gene family is naturally a critical factor in plant growth and development. 

*Panax ginseng* C. A. Meyer is a kind of slow-growing renascent herbs that belongs to the genus Ginseng in family *Araliaceae*. It has 4000 years of application history for its therapeutic effect in China according to an ancient Chinese medical book called Shen Nong’s herbal classic of materia medica. Panax is named after the ancient Greek πᾶν (pân, “all”) + ἄκος (ákos, “cure”). As the Greeks name describes, ginseng and its derived compounds have been proven to have therapeutic functions in a tremendous scope of diseases, such as cancer [20], cardiovascular disorders [21], autoimmune disease [22], rheumatic diseases [23], obesity [24], ocular diseases [25], and inflammation [25]. Besides, they have also been proven to be capable of improving strategic learning [26] and resistance to stress [27]. In spite of its excellent quality, ginseng is hard in cropping, owing to the disadvantages of its weak resistance to a lot of diseases, and especially, the long growth period. It takes at least four years for ginseng to become a medicinal material. Ginseng’s medicinal value increases along with its cultivation time, which explains why an illegal company may sell short-growing ginseng pretended to be long-growing ginseng in order to make money. The current method to judge the age of ginseng mainly relies on observing the appearance of ginseng, which is often not accurate enough. Therefore, there is an urgent need to develop a new way in judging the ginseng age through biotechnology.

In summary, the age judging of ginseng that *SPL* genes may be involving in was essential for application but have not been report to date. Hence, in this study, we identified *SPL* gene family from ginseng transcriptome for the first time, and then conducted the basic bioinformatics analysis including phylogenetic relationship, network interaction, and spatial and temporal expression patterns of it. These analysis will contribute to the exploration of the correlation between *SPL* gene expression and ginseng’s age. In addition, it provides a theoretical basis and bioinformatics database for ginseng genetics improvement and breeding in the future.

## 2. Materials and Methods

### 2.1. Identification of the Ginseng SPL Transcripts

Three approaches were applied to identify the ginseng *SPL* gene family in order to ensure its integrity. First, we performed the search for putative SBP amino acid sequences in ginseng from the Korean ginseng protein data (http://ginsengdb.snu.ac.kr/genome.php) taking the amino acid sequence of SBP domain (Pfam: PF03110) as the query. Then, the result was used as the query sequences for the TBLASTN to search from the Jilin ginseng transcriptome database for putative *SPL* transcripts in ginseng (Jilin *P. ginseng* transcriptome database was developed from 14 tissues of a 4-year-old Jilin ginseng plant, including fiber root, leg root, main root epiderm, main root cortex, rhizome, arm root, stem, leaf peduncle, leaflet pedicel, leaf blade, fruit peduncle, fruit pedicel, fruit flesh, and seed) at an e-value of 1 × 10^−6^. Second, nucleotide sequences of *SPL* in other species were downloaded from GenBank (http://www.ncbi.nlm.nih.gov/) and were used as the queries for the BLAST to search from the Chinese ginseng transcriptome database (http://ginseng.vicp.io:23488/) and Korean ginseng transcriptome database (http://ginsengdb.snu.ac.kr/transcriptome.php), at an e-value of 1 × 10^−6^. The results were used as the query sequences searching from the Jilin ginseng transcriptome database for putative *SPL* transcripts in ginseng at an e-value of 1 × 10^−6^. Third, the SBP amino acid sequence was downloaded from the Plant Transcription Factor Database (http://plntfdb.bio.uni-potsdam.de/v3.0/) as the query sequence for BLAST to search from the Jilin ginseng transcriptome database at an e-value of 1 × 10^−6^. Then, the results of the three approaches were merged and duplicates were removed. Finally, *PgSPL* gene sequences were submitted to ITAK (http://itak.feilab.net/cgi-bin/itak/index.cgi) to verify whether they contain the SBP domain.

### 2.2. GO (Gene Ontology) Annotation, Functional Categorization and Analyses

Since the multiple transcripts of a gene that generated by alternatively splicing are likely to have disparate biological functions [28], we used transcripts instead of genes to perform the GO annotation by Blast2GO [29]. We counted the number of *SPL* transcripts that were involved in a particular function as well as the number that was involved in multiple functions to show the gene ontology annotation of ginseng SPL gene family.

### 2.3. Multiple Sequence Alignments and Phylogenetic Analysis

In order to classify the *PgSPL* transcripts, we aligned *PgSPL* transcripts that have the complete SBP domain with seven other species. The sequences involving complete SBP domains were drawn out for phylogenetic analysis. Species belonging to dicotyledons and monocotyledons, especially belonging to asterid (which ginseng belongs to) were selected out as the outgroup species. Ten *SPL* transcripts in seven species were selected for sequence alignment and phylogenetic analysis, which consisted of *Arabidopsis thaliana*, *Antirrhinum majus*, *Daucus carota*, *Helianthus annuus*, *Lactuca sativa*, *Oryza sativa* subsp. Japonica and *Solanum lycopersicum*. The phylogenetic tree were conducted using the maximum-likelihood (ML) method of MEGA-X [30], and the bootstrap replications were set to 2000.

### 2.4. Ka/Ks Ratio among SPL in Ginseng and Other Species

According to the results of the previous step, *PgSPL*03 and *PgSPL*24-10 were selected to calculate the Ka/Ks ratio since their expression pattern was representative. The genes of other seven species previously used in the phylogenetic analysis were selected to calculate the Ka/Ks ratio. MEGA-X was used for the alignment and the YN00 of PAMLX software [31] was used for calculation of the Ka/Ks ratio.

### 2.5. Analysis of Different PgSPL Groups for Conserved Motifs

In order to gain a deeper insight into the evolution and classification of *PgSPL* transcripts, we conducted multiple sequence alignment using the MAFFT software within different groups that were obtained from the phylogenetic analysis. The results are shown by WebLogo [32,33] (http://weblogo.berkeley.edu/). The amino acids of SBP domain used in analysis were transformed from transcripts by NCBI ORF Finder (https://www.ncbi.nlm.nih.gov/orffinder/).

### 2.6. Expression and Interaction Characteristic of PgSPL Transcripts

In order to profile the expression pattern of *PgSPL* transcripts, we made heat map analysis by TBtools [34]. In order to profile the interaction characteristic among *PgSPL* transcripts, we calculated the Spearman’s rank correlation coefficients using the R language program R Core Team 2013 and the results were visualized as network by BioLayout Express 3D software 3.3 [35]. The expression of *PgSPL* in 14 different tissues of 4-year-old ginseng and 4 different growing-year (5-, 12-, 18-, 25-years) of ginseng roots were used to show the temporal and spatial characteristic of *PgSPL*, and the expression of 42 farmer’s cultivars of 4-year-old ginseng roots were used to show the characteristic within different genotypes.

## 3. Results

### 3.1. Identification of the Ginseng SPL Transcripts

To identify all *PgSPL* transcripts, we used three methods to search. Taking the SBP domain (Pfam:PF03110) as query, we obtained 478 transcripts; taking as queries the nucleotide sequence of SPL transcripts in other species, we obtained 209 transcripts; taking as query the SBP amino acid sequence downloaded from Plant Transcription Factor Database, we obtained 30,992 transcripts. Then, after consolidating the results of these three methods and removing the duplicates, we obtain 31,994 transcripts. Finally, to verify whether they had SBP domain, we submitted them to ITAK. A total of 106 sequences (Table 1) were verified as possessing the SBP domain. The sequences identified as including SBP were rechristened *PgSPL*01-*PgSPL*30 (Table S1), and transcripts of the same gene were distinguished by digital suffixes (e.g., −01).

### 3.2. GO Annotation of PgSPL Transcripts

**Figure 1:**
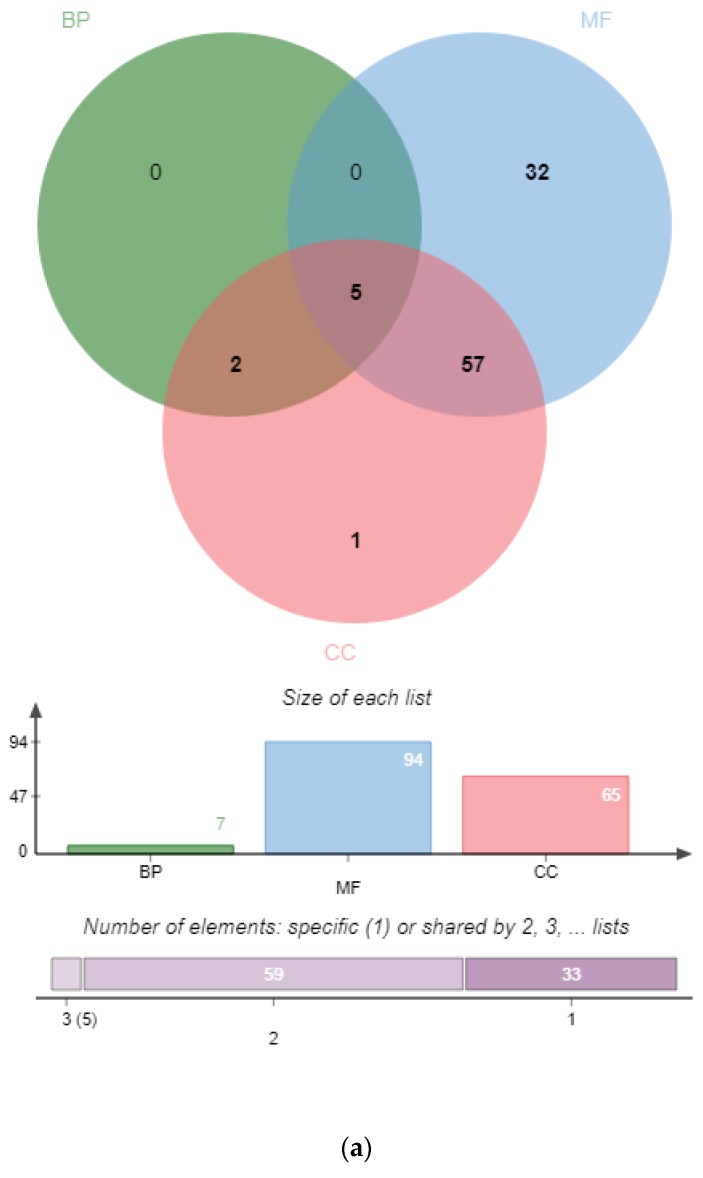

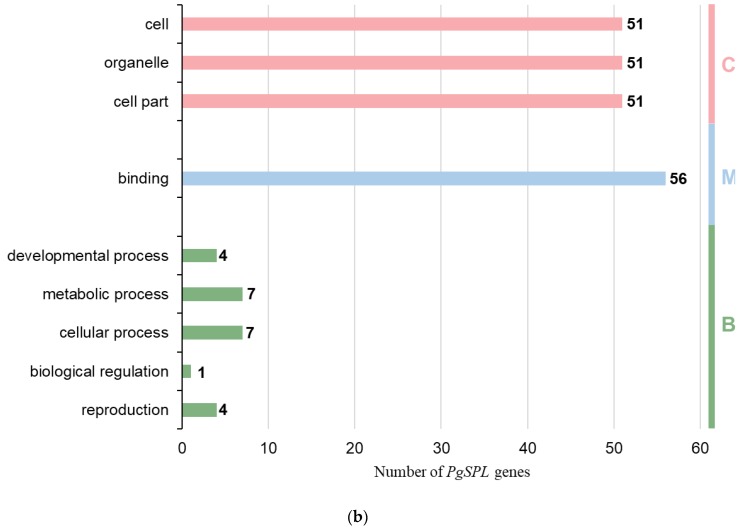
Functional categorization of the PgSPL transcripts by gene ontology (GO). (**a**) Venn diagram of the PgSPL transcripts among the biological process (BP), molecular function (MF) and cellular component (CC) functional categories. (**b**) The PgSPL transcripts are classified into 9 functional categories at Level 2, including three CC functional categories (Red), one MF functional category (Blue), and five BP functional categories (Green).

To provide an overview of functions of *PgSPL* transcripts, we annotated and categorized *PgSPL* transcripts using the free basic version of Blast2GO. The original annotation results of *PgSPL* are shown in Table S2. We further counted the number of transcripts that involved in different GO functions (Figure 1a), and the number that participate in multiple GO functions (Figure 1b). There were 99 of 106 *PgSPL* transcripts which have annotations in Blast2GO: 95 of them were annotated with Molecular Function, 70 of them were annotated with Cell Component, and only 14 were annotated with Biological Processes. Especially, there are 12 *PgSPL* transcripts annotated with all three functions above, which indicated the importance of them.

More concretely, the *PgSPL* transcripts were annotated with 16 specific GO annotations that belongs to the three functions above. The results are shown in Figure 1b. A total of 90 (91%) *PgSPL* transcripts were categorized to binding (GO:0005488); 56 (57%) were categorized to cell (GO:0005623), cell part (GO:0044464), and organelle (GO:0043226), respectively; and the amount of *PgSPL* transcripts that annotated with 12 other functions were no more than 20. This results indicated that the *PgSPL* gene family may involve in multiple functions, but the main function were focused on the binding function, including DNA binding (GO:0003677) and metal ion binding (GO:0046872). 

In this section, all *PgSPL* transcripts are expected to annotate with the “binding” function for their SBP domain. However, if we annotate all transcripts’ nucleotide sequences, only 56 transcripts will be annotated with the “binding” function. Thus, to explore whether it is the difference in open reading frames that caused the other 50 transcripts to be not annotated with “binding” function, we used open reading frames (ORFs) to annotate transcripts that were not annotated with the "binding" function in the first step. As the result, a total of 90 transcripts were annotated with the "binding" function. To clarify the reason why to some transcripts, only ORFs could be annotated with function while the complete nucleotide sequences could not, we separately calculated the ratio of the SBP domain sequence in the total sequence for the transcripts annotated with “binding” and for those were not annotated with “binding.” We found that the ratio of the former was 18% and the latter was 11%. Finally, to test if it was statistically significant, we used the SPSS software to independent-samples t-test these two set of ratio data, with the result showing that the difference between these two groups of ratios was extremely significant. On the basis of this, we concluded that it was their over length of complete nucleotide sequence that led to the fact that some transcripts nucleotide sequences could not be annotated with “binding” function. Given this problem, we annotated all *PgSPL* transcripts ORF and consolidated it with the annotation result of all *PgSPL* transcripts whole length nucleotides. A total of 16 transcripts still could not be annotated with the “binding” function. Part of them were not blasted and the rest were the result of an incompleteness of the SBP domain.

### 3.3. Phylogenetic Relationship and Classification for PgSPL Transcripts

**Figure 2:**
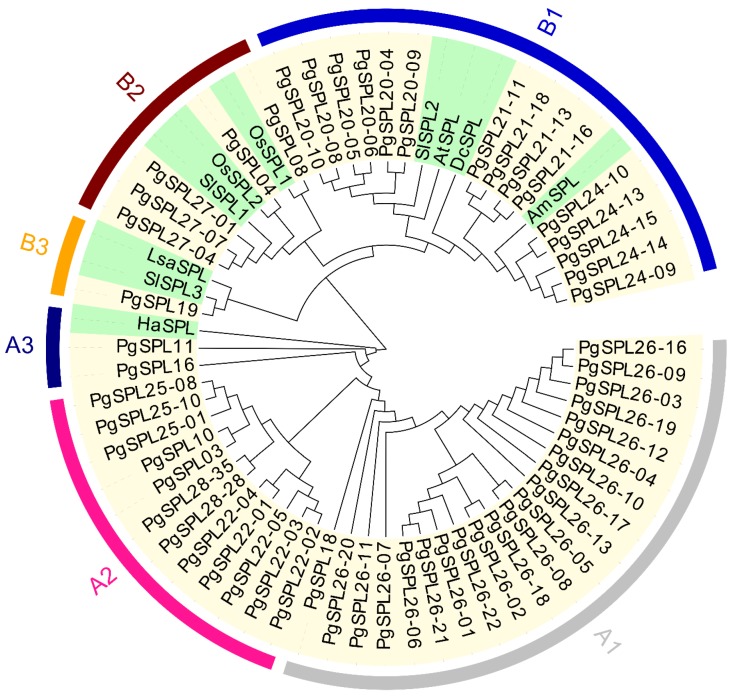
Phylogenetic tree of the *SPL* gene family in *Panax ginseng* and 7 other species. Yellowish in the inner circle indicates the *Panax ginseng* of *SPL* gene (*PgSPL*); Greenish in the inner circle indicates the *Arabidopsis thaliana* (*AtSPL*), *Antirrhinum majus* (*AmSPL*), *Daucus carota* (*DcSPL*), *Helianthus annuus* (*HaSPL*), *Lactuca sativa* (*LsSPL*), *Oryza sativa* subsp. Japonica (*OsSPL*) *and Solanum lycopersicum* (*SlSPL*) of *SPL* gene.

To obtain the phylogenetic relationship among *PgSPL* transcripts and *SPL* transcripts in other species, we chose 56 *PgSPL* transcripts with an intact SBP domain and ten transcripts from seven other species to construct a phylogenetic tree. The result is shown in Figure 2. The *SPL* transcripts are basically divided into two clades, A and B. Clades A and B respectively consisted of three groups, i.e., there were six groups in total, which was consistent with the present study [36]. All transcripts from *PgSPL*26 and *PgSPL*18 were classified into the A1 group. All transcripts of *PgSPL*22, 28 and 25 were classified into the A2 group. *PgSPL*11, 16 and *HaSPL* were classified into the A3 group. All transcripts of *PgSPL*20, 21, 24, *PgSPL*08, *SlSPL*2, *DcSPL*, *AtSPL,* and *AmSPL* were classified into the B1 group. All transcripts of *PgSPL*27, *PgSPL*04, *OsSPL*1, 2, and *SlSPL*1 were classified into the B2 group. *PgSPL*19, Sl*SPL*3, and *LsSPL* were classified into the B3 group (Figure 2; Table S3).

### 3.4. Synonymous and Nonsynonymous Substitution Rates and Selective Pressure in PgSPL

To explore the evolutionary pressure, we calculated the synonymous and nonsynonymous substitution rates (Ka/Ks) of *PgSPL* transcripts and compared them to the Ka/Ks ratio in other species. *PgSPL*03 and *PgSPL*24-10 were selected to calculate the Ka/Ks ratio using YN00 of PAMLX software. The result showed that all of the Ka/Ks ratios are lower than 1. To be specific, the average number is 0.25 with CV = 78%. The lowest one was 0.11 between *PgSPL03* and *Antirrhinum majus*, and the highest one was 0.88 between *PgSPL24-10* and *Daucus carota*. In ginseng, the Ka/Ks ratio between *PgSPL*03 and *PgSPL*24-10 was 0.21. Interestingly, *Daucus carota* is one of the species that have the closest phylogenetic relationship with ginseng, but its Ka/Ks ratios had a big difference with ginseng. If the *DcSPL* were excluded from the calculation, the average would be as low as 0.18 with a CV = 29%. All of these results indicated that *SPL* genes in eight species above had been undergoing a strong purifying selection.

### 3.5. Analysis for Conserved Domains of PgSPL Transcripts

**Figure 3:**
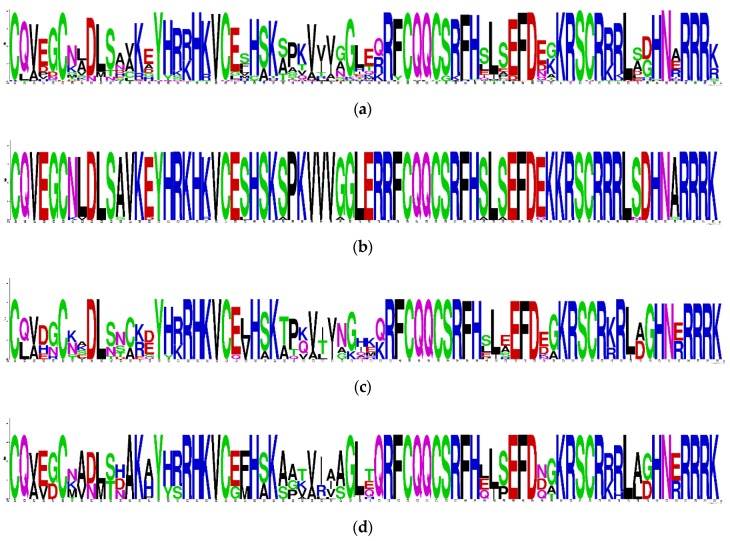

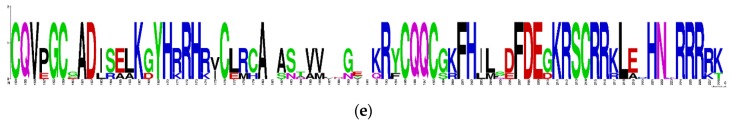
Conserved amino acid motifs in the SBP domain of the PgSPL proteins in ginseng. (**a**) Sequence logo of SBP domain of all SPL protein. (**b**) Sequence logo of SBP domain of A1 group. (**c**) Sequence logo of SBP domain of A2 group. (**d**) Sequence logo of SBP domain of B1 group. (**e**) Sequence logo of SBP domain of B2 group.

**Figure 4:**
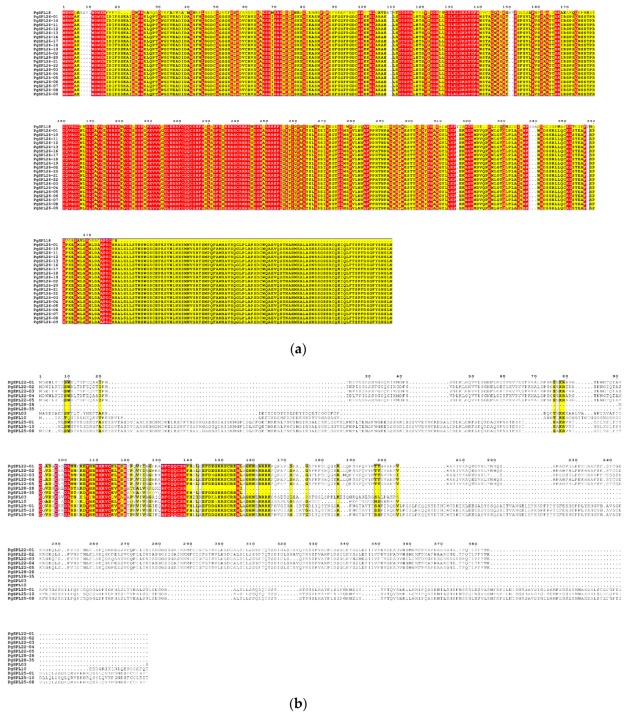

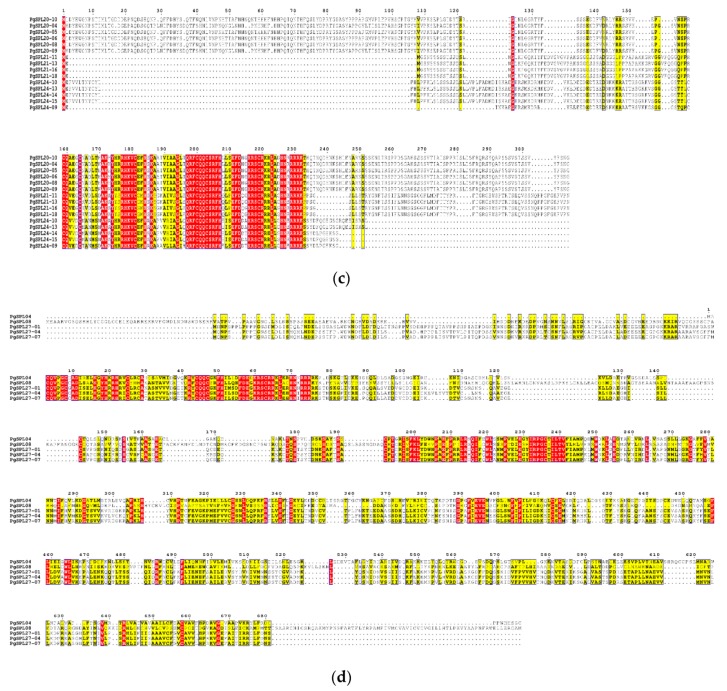
Multiple sequence alignment of SBP domain of the PgSPL proteins in ginseng. (**a**) Alignment of SBP domain of A1 group. (**b**) Alignment of SBP domain of A2 group. (**c**) Alignment of SBP domain of B1 group. (**d**) Alignment of SBP domain of B2 group.

To explore the structure difference of SBP domain among six different *PgSPL* transcript groups obtained from phylogenetic analysis, we conducted multiple sequence alignment using MAFFT software. In order to show the differences between groups more clearly, we used Weblogo to show the alignment result. Because of the deficiency in transcript numbers of group A3 and B3, we did not align these two. The result is shown in Figure 3 and Figure 4. Several amino acid in SBP domain were conserved in sequences of all groups, including: in the structure of zinc finger 1, the cysteine (C), cysteine (C), cysteine (C), and histidine (H) at position 214, 219, 236, and 239 amino acid, respectively; in the structure of zinc finger 2, the cysteine (C), cysteine (C), histidine (H), and cysteine (C) at position 255, 258, 262, and 274 amino acid, respectively; in the structure of nuclear localization signal (NLS), the lysine (K), arginine (R), arginine (R), arginine (R), arginine (R), and lysine (K) at position 271, 272, 284, 285, 286, and 287 amino acid, respectively. (Figure 3a). These conserved amino acid residues in *PgSPL* transcripts are basically consistent with group II reported in Guo’s study [37]. 

Within different groups, the conserved pattern of *PgSPL* sequences were different. In the group A1, all of the transcripts contained the C3HC2HC type SBP box with 74 amino acids in total and had a very strong consistency in the other positions apart from the two zinc fingers and the NLS (Figure 3b). The A2 group was also consistent with the two zinc fingers and an NLS with 74 amino acids in total. Its residues in the other positions apart from the three motifs, however, were not conserved so much (Figure 3c). As for the group B1, they also had a strong consistency in two zinc fingers and the NLS whose consistency was only lower than A1’s. The other positions of the group B1 apart from the three motifs were relatively conserved but differ from A1’s (Figure 3d). When it comes to the group B2, they had inferior consistency. Their SBP box was the C4C2HC type, which is quite another SBP box type from the C3HC2HC type SBP box conserved in all the rest of the *PgSPL* transcripts (Figure 3e). Because the conserved domains are essential for sequences to conduct functions, the difference of it in different groups may led to the functional differentiation among groups of *PgSPL*.

### 3.6. Temporal and Spatial Expression Characteristic of PgSPL Transcripts

**Figure 5:**
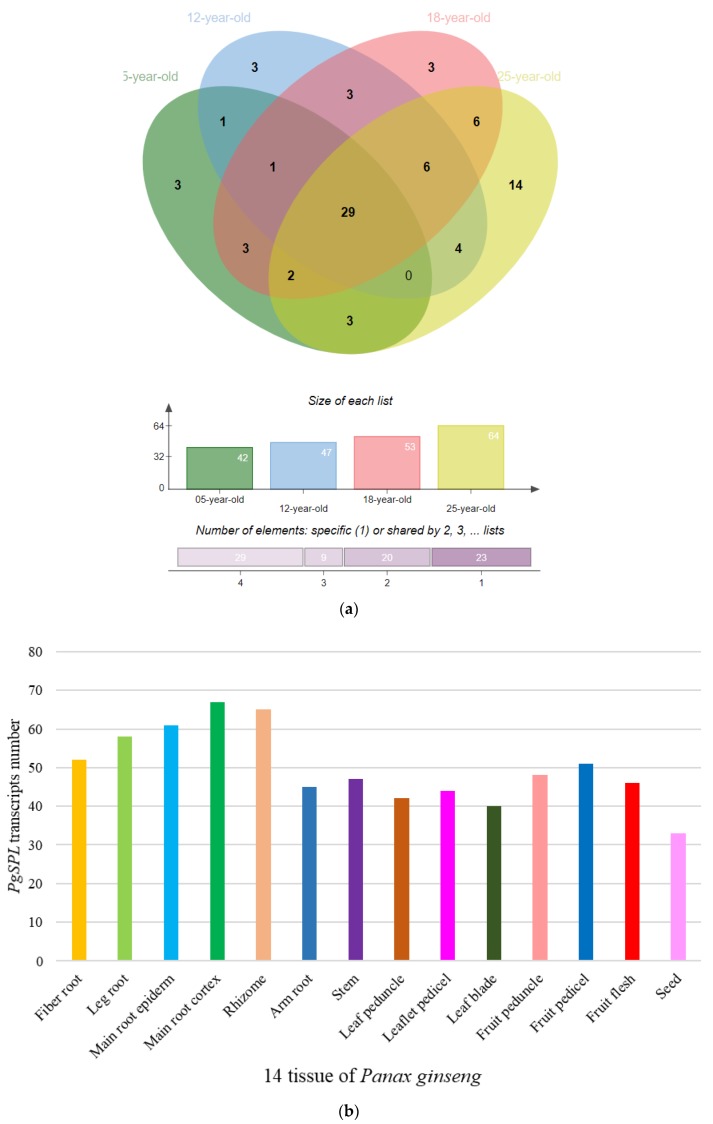
Proportion of expressed *PgSPL* transcripts in four different year-old roots groups and 14 tissues. (**a**) The Venn chart of the numbers of 81 *PgSPL* transcripts expressed in four age groups. (**b**) The histogram of 96 *PgSPL* transcripts expressed in 14 tissue.

In order to show temporal and spatial expression pattern of *PgSPL* transcripts, we conducted heat maps based on different growing years and different tissues. In different growing years of ginseng (5-, 12-, 18-, and 25-years), there were 81 *PgSPL* transcripts expressed out of total 106, and the number expressed in each growing year were 42, 47, 53, and 64 respectively. Obviously, the number of expressed *PgSPL* transcripts were increasing with the growing year longer of ginseng. In addition, only 29 (36%) transcripts expressed in all four growing years, most *PgSPL* transcripts expressed differential in different growing years. Especially, there are three (13%) transcripts expressed only in 5-year-old root, three (13%) only in 12-year-old root, three only in 18-year-old root. When it comes to 25-year-old root, however, the *PgSPL* transcripts specifically expressed in it went up to 14, accounting for 61% of total number of expressed transcripts (Figure 5a and Figure 8c).

In 14 tissues of the 4-year-old ginseng, a total of 96 expressed *PgSPL* transcripts out of 106 were used to construct the heat map (Figure 8a). Among them, 16 (17%) transcripts were expressed in all the tissues while nine (9.4%) transcripts specifically expressed in one tissue. Of these nine transcripts, five expressed only in fruit pedicel, two in leaflet pedicel, one in fruit peduncle, and one in the main root cortex. The rest of the 71 transcripts expressed in multiple tissues (Figure 5b).

**Figure 6:**
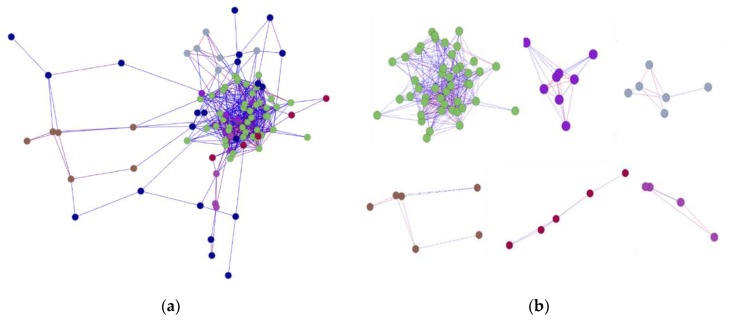

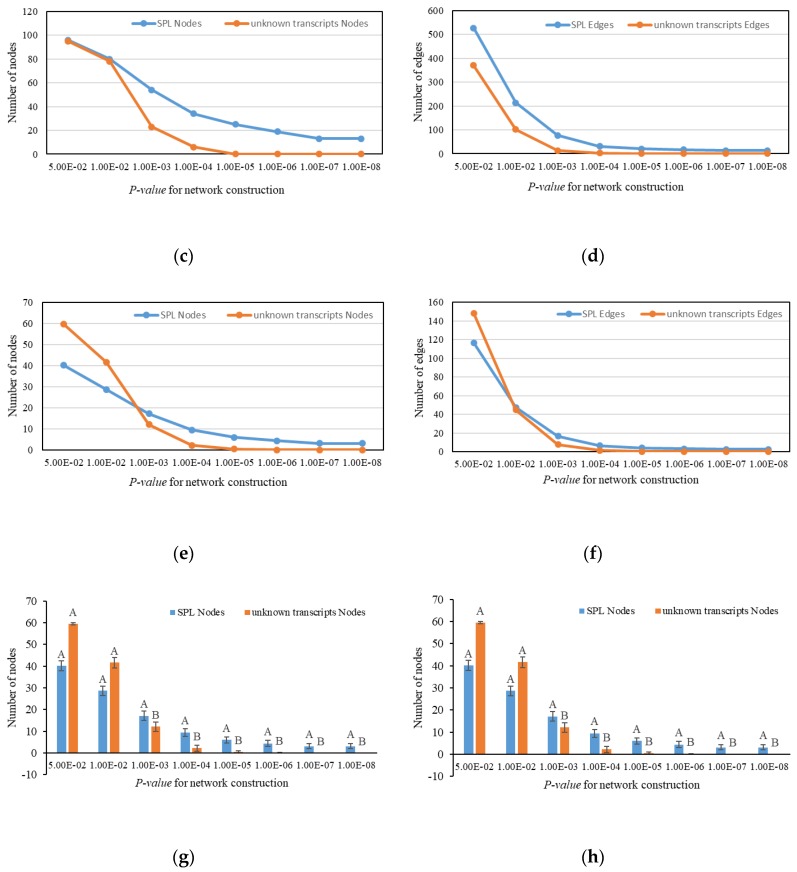
The network constructed at *p ≤* 0.05, and its analysis of the *PgSPL* transcripts expressed in the 14 tissue of 4 years old ginseng. (**a**) The network of all transcripts. (**b**) The networks of clusters. (**c**) Trend that numbers of nodes of all *PgSPL* transcripts under different *p*-values with unknown genes as controls. (**d**) Trend that numbers of edges of all *PgSPL* transcripts under different *p*-values with unknown genes as controls. (**e**) Trend that numbers of nodes of randomly drawn 64 *PgSPL* transcripts under different *p*-values with unknown genes as controls. (**f**) Trend that numbers of edges of randomly drawn 64 *PgSPL* transcripts under different *p*-values with unknown genes as controls. (**g**) Statistics of the numbers of nodes of randomly drawn 64 *PgSPL* transcripts under different *p*-values with unknown genes as controls. (**h**) Statistics of the numbers of nodes of randomly drawn 64 *PgSPL* transcripts under different *p*-values with unknown genes as controls. In (**g**) and (**h**), the error bars are standard deviation for 20 replications.

**Figure 7:**
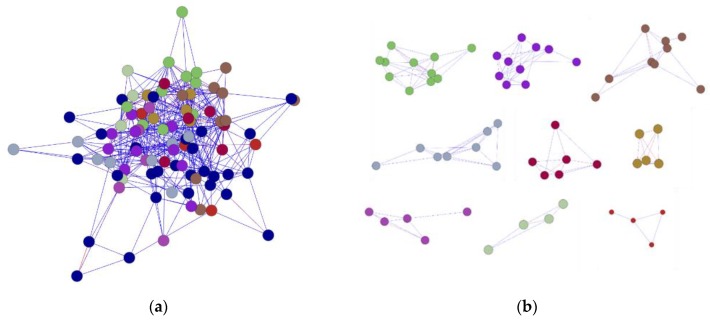

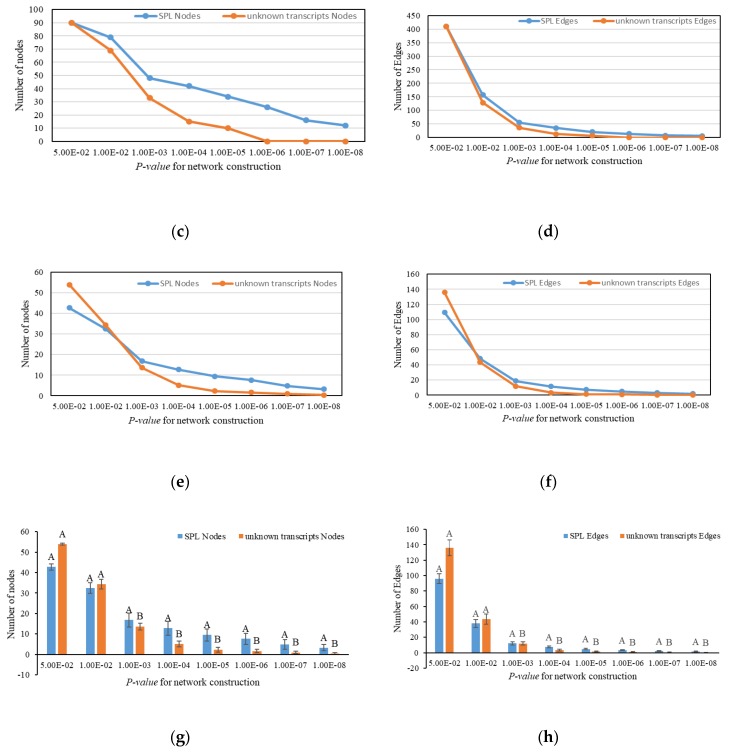
The network constructed at *p ≤* 0.05, and its analysis of the *PgSPL* transcripts expressed in the 42 farmers’ cultivars of 4 years old ginseng. (**a**) The network of all transcripts. (**b**) The networks of clusters. (**c**) Trend that numbers of nodes of all *PgSPL* transcripts under different *p*-values with unknown genes as controls. (**d**) Trend that numbers of edges of all *PgSPL* transcripts under different *p*-values with unknown genes as controls. (**e**) Trend that numbers of nodes of randomly drawn 60 *PgSPL* transcripts under different *p*-values with unknown genes as controls. (**f**) Trend that numbers of edges of randomly drawn 60 *PgSPL* transcripts under different *p*-values with unknown genes as controls. (**g**) Statistics of the numbers of nodes of randomly drawn 60 *PgSPL* transcripts under different *p*-values with unknown genes as controls. (**h**) Statistics of the numbers of nodes of randomly drawn 60 *PgSPL* transcripts under different *p*-values with unknown genes as controls. In (**g**) and (**h**), the error bars are standard deviation for 20 replications.

To show the spatial interaction characteristic of *PgSPL* transcripts, we calculated the Spearman’s rank correlation coefficients among all transcripts expressed in different tissues using the R language. Then, in order to make it easier to read, we visualized the results as a network by BioLayout Express 3D software. Under the *p*-value of 0.05, all *PgSPL* transcripts expressed in 14 tissues formed a single co-expression network (Figure 7a), which fell into six clusters (Figure 7b) and contained 96 nodes and 527 edges in total (Figure 6a). The average connectivity reached up to 10.979. In order to reflect the tightness of this network, we counted the nodes and edges of it under the increasingly strict *p*-value, and introduced same number of sequences randomly drawn from ginseng transcriptome to construct network as the negative control. The results were showed in Figure 6c,d. Under *p =* 0.05, the number of nodes and edges of all *SPL* transcripts were 96 and 527 respectively; and for negative control were 95 and 370, respectively. However, under the *p =* 1 × 10^−5^, the number of nodes and edges of all *SPL* transcripts were 25 and 21 respectively, while that of nodes and edges of unknown transcripts both were 0. This result indicated that the *PgSPL* transcripts formed a tighter network than the randomly drawn transcripts. To further prove the correlation between the pairs of each *SPL* transcripts, we constructed the network using randomly drawn two-thirds of total *SPL* transcripts (which was 64) and introduced the negative control as described above (Figure 6e,f). After 20 repetitions, we calculated the average of nodes and edges respectively. The result are shown in Figure 6g,h. Under the *p =* 0.05, the average number of nodes and edges of random 64 *SPL* transcripts were 40.1 and 116.65 respectively; the average number of nodes and ages of 64 random unknown transcripts were 59.5 and 148.3, respectively. Under the *p =* 1 × 10^−8^, the number of nodes and edges of 64 *SPL* transcripts were 3.1 and 2.45, respectively, while that of nodes and edges of 64 unknown transcripts were both 0. Especially, the average number of nodes and edges of *PgSPL* network were significantly larger than that of the random unknown genes under the *p* = 1 × 10^−3^ to *p* = 1 × 10^−8^. These results indicated that the *PgSPL* transcripts have a significant co-expression interaction with each other and might reflect the mechanism underlying the *PgSPL* functions.

### 3.7. Expression Characteristic of PgSPL Transcripts in 4-Year-Old Roots of Different Genotypes

We explored the expression pattern of *PgSPL* in different genotype through the heat map analysis of *PgSPL* transcripts in 4-year-old roots of 42 farmers’ cultivars. A total of 90 of 106 transcripts expressed in the roots of the 42 farmer’s cultivar. The CV of the number of expressed transcripts in every genotype’s roots was 11%, which indicated that the expression of *PgSPL* transcripts has consistency in different genotypes. 

To show the correlation among all *PgSPL* transcripts in different genotypes, we calculated Spearman’s rank correlation coefficients among all transcripts expressed in different genotypes using the R language. Then, in order to make it easier to read, we visualized the results using the BioLayout Express 3D software. Under the *p =* 0.05, the network of expressed transcripts in 42 farmer’s cultivars fell into nine clusters and consisted of 90 nodes and 409 edges in total (Figure 7b). The average connectivity of the network was 9.089. In order to reflect the tightness of this network, we counted the nodes and edges of it under the increasingly strict *p*-value, and introduced same number of sequences randomly drawn from ginseng transcriptome to construct network as the negative control. The results are shown in Figure 7c,d. Under *p* = 0.05, both the *SPL* transcripts’ and unknown transcripts’ number of nodes and edges were respectively 90 and 409. However, under the *p =* 1 × 10^−6^, the number of nodes and edges of all *SPL* transcripts are 26 and 14 respectively, while negative control’s nodes and edges both were 0 (Figure 7a–d). This result indicated that the *PgSPL* transcripts formed a tighter network than the randomly drawn transcripts. To further prove the correlation between pairs of each *SPL* transcripts, we constructed the network using randomly drawn two-thirds of total *SPL* transcripts (which was 60) and introduced the negative control as described above (Figure 7e,f). After 20 repetitions, we calculated the average of them. The result shows that under the *p =* 0.05, the average number of nodes and edges of random 60 SPL transcripts were 42.7 and 109.2, respectively; the average number of nodes and ages of 60 random unknown transcripts were 53.9 and 135.85, respectively. However, under the *p* = 1 × 10^−8^, the number of nodes and edges of 60 SPL transcripts were 3.25 and 2, respectively, while negative control’s nodes and edges were 0.4 and 0.2, respectively (Figure 7e–h). This was the same as the network of expressed *SPL* transcripts in 14 tissues. The network of expressed *SPL* transcripts in 42 farmers’ cultivars pointed to the fact that the expression of some *SPL* transcripts in different tissues had a close correlation when performing their biological functions.

**Figure 8:**
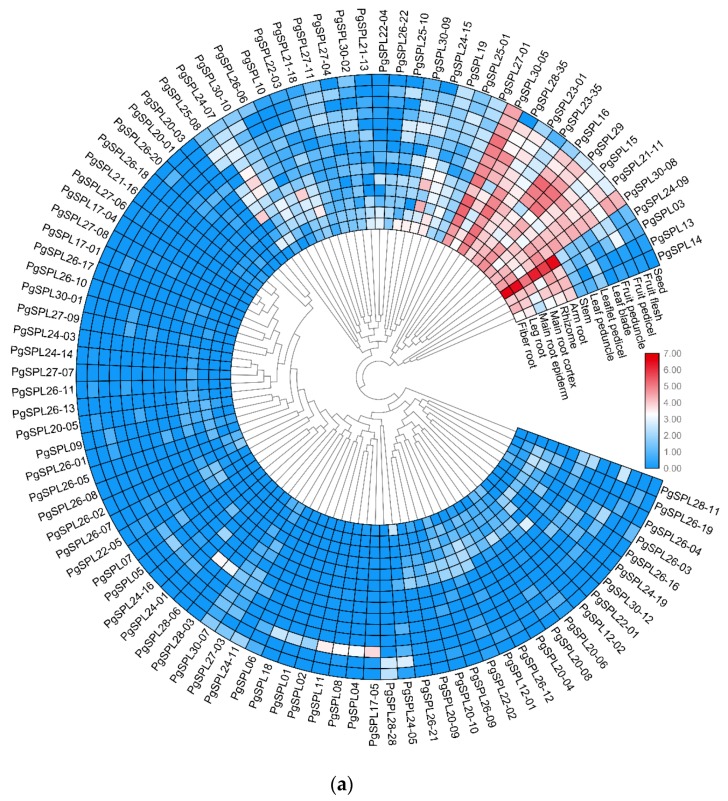

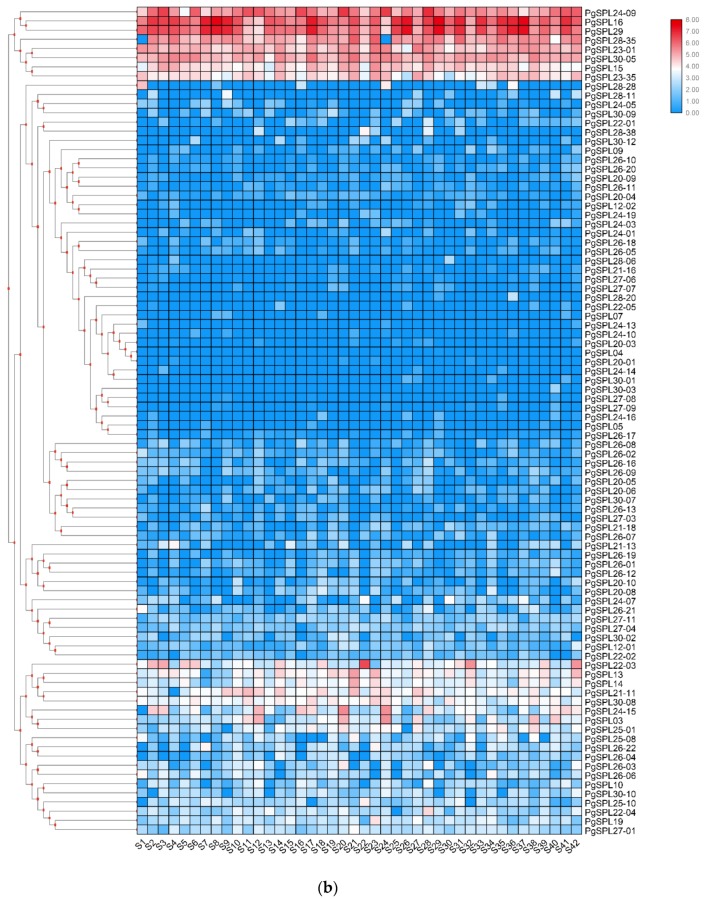

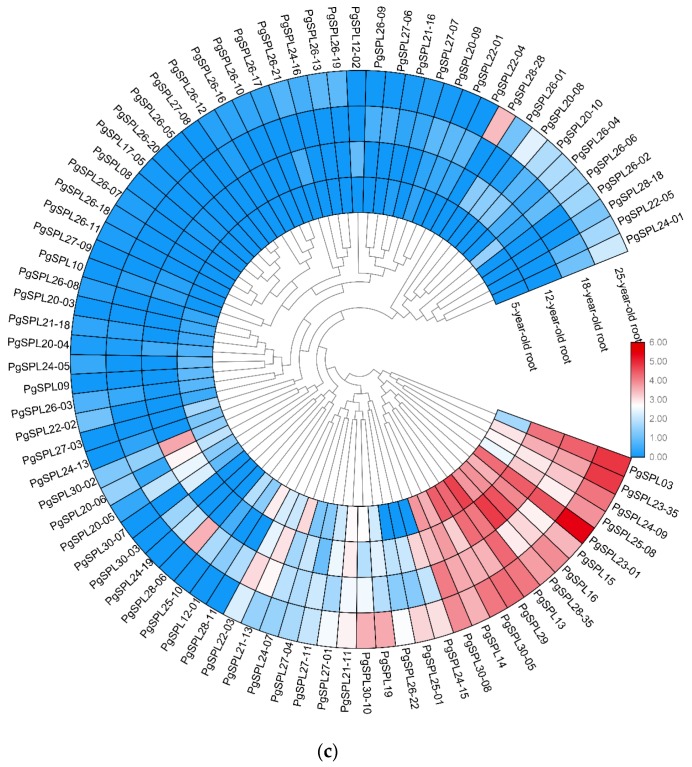
Expression heatmaps of the *PgSPL* gene family transcripts in Jilin ginseng. (**a**) The 106 *PgSPL* transcripts expressed in the 14 tissue of 4 years old ginseng. (**b**) The 106 *PgSPL* transcripts expressed in the 42 genotypes of 4 years old ginseng. (**c**) The 106 *PgSPL* transcripts expressed in the four different years old ginseng.

To show the expression pattern in different genotypes, we visualized the expression quantity data of 42 farmer’s cultivars by TBtools [34]. From the heat map, we could see that the expression quantity distributions of *PgSPL*03, 13, 14, 21-11, 22-03, 24-15, 28-35, and 30-08 were very outstanding, among which *PgSPL*28-35 had a high expression quantity in almost all genotype except for S1 and S12, while the rest of these had low expression quantities in most genotype except for several farmers’ cultivars (Figure 8b; Table S4).

## 4. Discussion

### 4.1. SPL Genes in Ginseng is a Biggish Gene Family

Chlamydomonas reinhardtii, Physcomitrella patens, Selaginella moellendorffii, Pinus taeda, Picea glauca, Arabidopsis thaliana, Populus trichocarpa, Oryza sativa, and Zea mays respectively have 7, 14, 13, 3, 2, 16, 26, 18, and 21 SPL genes [37]. The average number in these nine species is 13.3, which is significantly lower than the SPL in Panax ginseng whose number is 30.

### 4.2. PgSPL Transcripts Have a Relatively Centralized Distribution of Functions and Tend to Serve for Close Purposes

According to the present studies, *SPL* transcripts are associated with biological processes such as regulation for flowering [8,9], and molecular function such as DNA-binding [16]. Hence, most of them should have been annotated with biological processes and molecular functions. The GO result shows that, however, only 14 (14%) transcripts are annotated with a biological process, while 70 (71%) transcripts are annotated with cell components, which indicates that the function of SPL in ginseng quite differs from the SPL in other species. SPL transcripts in other species are mostly regulatory genes that control the plant development process, while in ginseng, they are not only regulatory genes but also structural genes. A total of 95 (96%) and 70 (71%) transcripts are categorized into molecular functions and cell components, respectively. To be specific, 90 (91%), 56 (57%), 56 (57%) and 56 (57%) transcripts are categorized into the binding, cell, organelle, and cell part, respectively. With SBP domains, all SPL transcripts are expected to basically associate with DNA-binding. Nonetheless, the heat maps show that the difference of *PgSPL* transcripts’ expressions in different tissues and age groups are very large and pronounced, indicating that certain *PgSPL* transcripts can just be expressed in a single tissue or a specific development stage (Figure 8a,c); furthermore, some of the *SPL* transcripts are target genes of miR156/157 and the rest of them are not [14]. All the results above point to the fact that *PgSPL* transcripts have similar functions and perform their functions through a single system.

### 4.3. Some PgSPL Transcripts with a Prominent Spatial Expression Pattern

From the angle of spatial expression pattern, *PgSPL*03, 13, 14, 23-01, 23-35, 24-09 and 24-15 tend to be expressed in underground parts of ginseng rather than the aboveground parts, among which the tendencies of *PgSPL*03, 13, and 14 are particularly obvious. On the contrary, the *PgSPL*16, 28-35 and 29 tend to be expressed in the aboveground parts such as leaf, rather than underground parts (Figure 8a; Table S4). It is obvious that *PgSPL* transcripts specifically expressed in underground parts outnumber those specifically expressed in the aboveground parts, which indicates that *PgSPL* transcripts play a crucial role more in the roots instead of other tissues such as leaf or seed.

### 4.4. Some PgSPL Transcripts with a Prominent Temporal Expression Pattern

It is reported that the expression of miR156 is regulated by age. To be specific, miR156 is highly accumulated in seedlings and gradually decreases as the plant ages in *Arabidopsis thaliana* [18,38]. The sense of the age of miR156 is prevalent in different species. In maize, rice, and poplar, the expression of miR156 decreases as age increases [5,39,40]. This result suggests that the upstream of miR156 may be a very conservative age signal of the age pathway and content of miR156 determines the physiological age of the plant [19]. Overexpression of miR156 prolongs the plant’s infancy, with increases in branching and leaf growth as well as delays of the flowering phenotype. These phenotypes are observed in Arabidopsis [8,18,38], maize [39], rice [5], tomato [13], and poplar [40]. Conversely, inhibition of miR156 activity by "target mimicry" in Arabidopsis can promote plants into adulthood [18,38,41,42].

The miR172 is also an important molecule that regulates plant transformation from childhood to adulthood [18]. Contrary to the expression pattern of miR156, the expression level of miR172 increases as age increases. Experiments with chromatin immunoprecipitation have shown that miR172 is a direct *SPL* downstream gene. In Arabidopsis, *SPL*9 and *SPL*10 bind directly to the promoter of miR172B and activate its transcription [18]. In miR156 target mimicry transgenic plants, the content of miR172 increases [18]. The function of miR172 in plants is opposite to miR156. Overexpression of miR172 causes Arabidopsis to enter adulthood in advance [8].

Thus, according to the present studies, *SPL* gene expression should go up with the decrease of miR156 and the increase of miR157 expression. Eventually, the plant transition is activated and moves from the vegetative process to the reproductive process. In our study, *PgSPL*03, 23-01, 23-35, 24-09, and 25-08 have a distinct tendency of expression increasing over time, which is consistent with the present studies. However, this is not a story of a uniform increase of all *SPL* transcripts. In other words, not all the expression pattern of *PgSPL* transcripts are consistent with the present studies. The expressions of *PgSPL*24-07 and 28-11 even decrease over time. *PgSPL*19, 22-04, 25-01, 30-10 barely express in the roots of the first three age groups (5, 12, and 18 years old), instead of only expressing in 25 years old roots. *PgSPL*30-02 and *PgSPL* 28-06 express only in 12-year-old and 18-year-old roots, respectively. One can notice that *PgSPL*30-02 and *PgSPL*30-10 come from the same gene, *PgSPL*30. However, they have entirely different temporal expressions, so do *PgSPL*24 and 28. Another noticeable point is that some *PgSPL* transcripts such as *PgSPL*15, 16, and 28-35 almost have a high expression in all ages of roots except those in 18-year-old roots (Figure 8c; Table S4). A total of 42 (52%), 47 (58%), 53 (65%), and 64 (79%) of transcripts express in 5, 12, 18, and 25 year-old roots respectively. To be specific, 46 (57%) of the transcripts have a higher expression level in 25-year-old roots than those in 5-year-old roots, and 26 (32%) of the transcripts have a lower expression in 25-years-old roots than those in 5-year-old roots, which is just partly consistent with the present studies. In a nutshell, the expression pattern of *SPL* transcripts in ginseng is very complicated and not completely consistent with the *SPL* transcripts in other species.

### 4.5. Some PgSPL Transcripts with a Prominent Genotype Expression Pattern

From the expressions of *PgSPL* transcripts in different genotypes, most transcripts’ expressions are very close in all farmers’ cultivars, which indicates that the expression of *PgSPL* transcripts has a universal characteristic. However, there are also specific characteristics of *PgSPL*. *PgSPL*28-35 expresses in almost all the farmers’ cultivars except S1 and S24, and *PgSPL*22-03 specifically has an intense expression in S22 (Figure 8b; Table S4).

### 4.6. PgSPL as Ginseng’s Age Molecular Markers

From the temporal expression pattern perspective, we found that the number of expressed *PgSPL* transcripts were increasing with the growing year longer of ginseng and most *PgSPL* transcripts expressed differential in different growing years. The expression of *PgSPL03*, *PgSPL23-35*, *PgSPL24-09*, *PgSPL25-08,* and *PgSPL23-01* had an obvious tendency to increase with age. Additionally, from the expression heat maps of 14 tissues and 42 genotypes, we found that *PgSPL23-35* and *PgSPL24-09* expressed steadily in all genotypic ginseng’ roots. Therefore, if the relationship between these two transcripts’ expression and ginseng’s age are verified and a standard curve is drawn, they can act as ginseng age’s molecular marker.

## 5. Conclusions

In this study, 106 *PgSPL* transcripts were identified. According to the annotation results, we found that *PgSPL* gene family might be involved in multiple functions, but the main function is focused on the binding function. We calculated the synonymous and nonsynonymous substitution rates and the results indicated that *SPL* genes had been undergoing a strong purifying selection. Through phylogenetic analysis, we found that *SPL* transcripts were basically divided into two clades. These two clades respectively consisted of three groups, which was to say, there were six groups in total. On the basis of this, we aligned the SBP domain for each groups. The results showed that both the C3HC2HC type SBP and C4C2HC type SBP existed in ginseng. We constructed the network of *PgSPL* transcripts expressed in 14 tissues and 42 genotypes and did the network analyses. The results indicated that the *PgSPL* transcripts have a significant co-expression interaction with each other and might reflect the mechanism underlying the *PgSPL* functions. According to *PgSPL* transcripts’ expression characteristics, we found that *PgSPL23-35* and *PgSPL24-09* were the most proper two transcripts to further study as ginseng age’s molecular marker. 

## Figures and Tables

**Table 1:** *PgSPL* gene family.

Gene	Transcript	Seq. Length (bp)	ORF Length (aa)	MW (kDa)	pI
*PgSPL01*	*PgSPL01*	231	65	7.22	8.79
*PgSPL02*	*PgSPL02*	529	88	10.08	4.81
*PgSPL03*	*PgSPL03*	988	182	20.32	8.74
*PgSPL04*	*PgSPL04*	2342	692	77.39	6.76
*PgSPL05*	*PgSPL05*	280	76	9.28	10.73
*PgSPL06*	*PgSPL06*	254	76	8.83	9.84
*PgSPL07*	*PgSPL07*	216	58	6.53	8.88
*PgSPL08*	*PgSPL08*	3216	961	105.47	5.67
*PgSPL09*	*PgSPL09*	264	81	8.81	9.64
*PgSPL10*	*PgSPL10*	1037	199	21.92	8.83
*PgSPL11*	*PgSPL11*	3582	966	106.26	6.82
*PgSPL12*	*PgSPL12-01*	511	63	6.56	4.58
	*PgSPL12-02*	523	66	6.80	4.58
*PgSPL13*	*PgSPL13*	832	74	8.56	11.40
*PgSPL14*	*PgSPL14*	1097	210	22.96	9.03
*PgSPL15*	*PgSPL15*	439	67	7.28	4.33
*PgSPL16*	*PgSPL16*	930	198	22.16	8.91
*PgSPL17*	*PgSPL17-01*	4180	264	29.47	6.25
	*PgSPL17-04*	4149	307	35.33	6.19
	*PgSPL17-05*	3882	264	29.47	6.25
*PgSPL18*	*PgSPL18*	1146	378	41.28	9.52
*PgSPL19*	*PgSPL19*	684	182	20.82	9.77
*PgSPL20*	*PgSPL20-01*	982	65	7.47	5.68
	*PgSPL20-10*	994	307	34.87	8.66
	*PgSPL20-03*	1779	65	7.47	5.68
	*PgSPL20-04*	1697	307	34.33	8.72
	*PgSPL20-05*	1685	307	34.87	8.66
	*PgSPL20-06*	1786	307	34.87	8.66
	*PgSPL20-08*	1791	307	34.87	8.66
	*PgSPL20-09*	1798	307	34.33	8.72
*PgSPL21*	*PgSPL21-11*	2176	213	22.94	9.78
	*PgSPL21-13*	2164	213	22.94	9.78
	*PgSPL21-16*	2206	213	22.94	9.78
	*PgSPL21-18*	2218	213	22.94	9.78
*PgSPL22*	*PgSPL22-01*	1827	384	42.24	8.26
	*PgSPL22-02*	1751	384	42.25	8.01
	*PgSPL22-03*	1691	380	41.86	7.61
	*PgSPL22-04*	1827	384	42.24	8.26
	*PgSPL22-05*	1735	384	42.26	8.26
*PgSPL23*	*PgSPL23-01*	1046	247	27.33	7.64
	*PgSPL23-35*	454	70	7.95	9.50
*PgSPL24*	*PgSPL24-01*	531	68	7.79	6.79
	*PgSPL24-10*	1312	186	21.35	8.77
	*PgSPL24-11*	1968	126	14.21	6.95
	*PgSPL24-13*	1211	186	21.35	8.77
	*PgSPL24-14*	776	177	20.27	8.76
	*PgSPL24-15*	1783	177	20.25	8.77
	*PgSPL24-16*	680	95	11.58	9.35
	*PgSPL24-19*	1026	68	7.79	6.79
	*PgSPL24-03*	1155	61	6.87	9.94
	*PgSPL24-05*	1127	95	11.58	9.35
	*PgSPL24-07*	1187	58	6.68	10.23
	*PgSPL24-09*	430	136	15.59	8.68
*PgSPL25*	*PgSPL25-01*	2764	533	58.86	8.29
	*PgSPL25-10*	2408	533	58.86	8.29
	*PgSPL25-08*	2606	537	59.32	8.10
*PgSPL26*	*PgSPL26-01*	2025	469	50.92	8.14
	*PgSPL26-10*	2377	469	50.90	8.14
	*PgSPL26-11*	1844	469	50.90	8.14
	*PgSPL26-12*	1932	469	50.90	8.14
	*PgSPL26-13*	2484	469	50.90	8.14
	*PgSPL26-16*	2044	469	50.90	8.14
	*PgSPL26-17*	2391	469	50.90	8.14
	*PgSPL26-18*	2591	469	50.92	8.14
	*PgSPL26-19*	1860	469	50.90	8.14
	*PgSPL26-02*	2288	469	50.92	8.14
	*PgSPL26-20*	2407	469	50.90	8.14
	*PgSPL26-21*	1948	469	50.92	8.14
	*PgSPL26-22*	2177	469	50.92	8.14
	*PgSPL26-03*	1955	469	50.90	8.14
	*PgSPL26-04*	2193	469	50.90	8.14
	*PgSPL26-05*	2270	469	50.92	8.14
	*PgSPL26-06*	2043	469	50.92	8.14
	*PgSPL26-07*	2502	469	50.90	8.14
	*PgSPL26-08*	1937	469	50.92	8.14
	*PgSPL26-09*	2132	469	50.90	8.14
*PgSPL27*	*PgSPL27-01*	3029	795	88.78	6.53
	*PgSPL27-11*	2821	446	50.05	7.56
	*PgSPL27-03*	2879	447	50.07	7.57
	*PgSPL27-04*	2886	791	88.44	6.61
	*PgSPL27-06*	2864	446	50.05	7.56
	*PgSPL27-07*	2828	786	87.97	6.32
	*PgSPL27-08*	2806	446	50.05	7.56
	*PgSPL27-09*	3051	446	50.05	7.56
*PgSPL28*	*PgSPL28-11*	467	110	12.45	8.97
	*PgSPL28-13*	614	83	9.54	10.51
	*PgSPL28-18*	509	247	27.25	5.74
	*PgSPL28-19*	480	120	13.28	8.97
	*PgSPL28-20*	1085	249	27.64	6.32
	*PgSPL28-27*	680	120	13.28	8.97
	*PgSPL28-28*	1092	50	5.90	9.40
	*PgSPL28-03*	693	72	8.27	5.25
	*PgSPL28-30*	509	111	12.48	8.97
	*PgSPL28-35*	575	65	7.84	9.42
	*PgSPL28-38*	538	111	12.48	8.97
	*PgSPL28-06*	588	119	13.25	8.97
*PgSPL29*	*PgSPL29*	3288	828	91.11	6.17
*PgSPL30*	*PgSPL30-01*	1106	157	17.03	5.68
	*PgSPL30-10*	878	113	12.89	8.47
	*PgSPL30-12*	695	104	11.25	5.40
	*PgSPL30-02*	1631	113	12.89	8.47
	*PgSPL30-03*	1414	90	10.33	9.10
	*PgSPL30-05*	1328	210	22.32	5.79
	*PgSPL30-07*	1162	210	22.32	5.79
	*PgSPL30-08*	998	157	17.03	5.68
	*PgSPL30-09*	859	157	16.54	5.51

## Supplementary Materials

The following are available online at https://www.mdpi.com/2223-7747/9/3/354/s1. Table S1: The nucleic acid sequences and protein sequences of the PgSPL gene transcripts. Table S2: The classification, annotation, and GO functional categorization of the *PgSPL* gene transcripts. Table S3: The identified genes used as evolutionary controls for *PgSPL* genes phylogenetic analysis. Table S4: The expressions of *PgSPL* transcripts (TPM) in 14 tissues, 42 farmers’ cultivars, and 4 different years old ginseng.

## Additional Information

The sequences of the *PgSPL* genes will be submitted to NCBI under BioProject RJNA302556.

## References

[1] Luscombe N.M., Austin S.E., Berman H.M., Thornton J.M. (2000). An overview of the structures of protein–DNA complexes. Genome Biol..

[2] Klein J., Saedler H., Huijser P. (1996). A new family of DNA binding proteins includes putative transcriptional regulators of the *Antirrhinum majus* floral meristem identity gene *SQUAMOSA*. Mol. Gen. Genet..

[3] Riese M., Zobell O., Saedler H., Huijser P. (2008). SBP-domain transcription factors as possible effectors of cryptochrome-mediated blue light signalling in the moss *Physcomitrella patens*. Planta.

[4] Cardon G.H., Höhmann S., Nettesheim K., Saedler H., Huijser P. (1997). Functional analysis of the *Arabidopsis thaliana* SBP-box gene *SPL*3: A novel gene involved in the floral transition. Plant J..

[5] Xie K., Wu C., Xiong L. (2006). Genomic organization, differential expression, and interaction of *SQUAMOSA* promoter-binding-like transcription factors and microRNA156 in rice. Plant Physiol..

[6] Salinas M., Xing S., Höhmann S., Berndtgen R., Huijser P. (2012). Genomic organization phylogenetic comparison and differential expression of the SBP-box family of transcription factors in tomato. Planta.

[7] Shao F.J., Lu Q., Wilson I.W., Qiu D.Y. (2017). Genome-wide identification and characterization of the *SPL* gene family in *Ziziphus jujube*. Gene.

[8] Wu G., Poethig R.S. (2006). Temporal regulation of shoot development in *Arabidopsis thaliana* by miR156 and its target *SPL*3. Development.

[9] Gandikota M., Birkenbihl R.P., Höhmann S., Cardon G.H., Saedler H., Huijser P. (2007). The miRNA156/157 recognition element in the 3′ UTR of the Arabidopsis SBP box gene *SPL*3 prevents early flowering by translational inhibition in seedlings. Plant J..

[10] Unte U.S., Sorensen A.M., Pesaresi P., Gandikota M., Leister D., Saedler H., Huijser P. (2003). *SPL*8, an SBP-box gene that affects pollen sac development in Arabidopsis. Plant Cell.

[11] Schwarz S., Grande A.V., Bujdoso N., Saedler H., Huijser P. (2008). The microRNA regulated SBP-box genes *SPL*9 and *SPL*15 control shoot maturation in Arabidopsis. Plant Mol. Biol..

[12] Wang J.W., Schwab R., Czech B., Mica E., Weigel D. (2008). Dual effects of miR156-targeted *SPL* transcripts and CYP78A5/KLUH on plastochron length and organ size in *Arabidopsis thaliana*. Plant Cell.

[13] Zhang X., Zou Z., Zhang J., Zhang Y., Han Q., Hu T., Xu X., Liu H., Li H., Ye Z. (2011). Over-expression of sly-miR156a in tomato results in multiple vegetative and reproductive trait alterations and partial phenocopy of the *sft* mutant. FEBS Lett..

[14] Xing S., Salinas M., Hohmann S., Berndtgen R., Huijser P. (2010). miR156-targeted and nontargeted SBP-Box transcription factors act in concert to secure male fertility in Arabidopsis. Plant Cell.

[15] Zhang Y., Schwarz S., Saedler H., Huijser P. (2007). *SPL*8, a local regulator in a subset of gibberellin-mediated developmental processes in Arabidopsis. Plant Mol. Biol..

[16] Bikenbihl R.P., Jach G., Saedler H., Huijser P. (2005). Functional dissection of the plant-specific SBP-domain: Overlap of the DNA-binding and nuclear localization domains. J. Mol. Biol..

[17] Rhoades M.W., Reinhart B.J., Lim L.P., Burge C.B., Bartel B., Bartel D.P. (2002). Prediction of plant microRNA targets. Cell.

[18] Wu G., Park M.Y., Conway S.R., Wang J.W., Weigel D., Poethig R.S. (2009). The sequential action of miR156 and miR172 regulates developmental timing in Arabidopsis. Cell.

[19] Yu S., Wang J.W. (2014). Recent Progress in miR156-mediated aging pathway in plants. Chin. Sci. Bull..

[20] Ahuja A., Kim J.H., Kim J.-H., Yi Y.-S., Cho J.Y. (2018). Functional role of ginseng-derived compounds in cancer. J. Ginseng Res..

[21] Irfan M., Kim M., Rhee M.H. (2019). Anti-platelet role of Korean ginseng and ginsenosides in cardiovascular diseases. J. Ginseng Res..

[22] Lee J.-I., Park K.S., Cho I.-H. (2019). *Panax ginseng*: A candidate herbal medicine for autoimmune disease. J. Ginseng Res..

[23] Yi Y.-S. (2019). Ameliorative effects of ginseng and ginsenosides on rheumatic diseases. J. Ginseng Res..

[24] Zhou S.S., Auyeung K.K., Yip K.M., Ye R., Zhao Z.Z., Mao Q., Xu J., Chen H.B., Li S.L. (2018). Stronger anti-obesity effect of white ginseng over red ginseng and the potential mechanisms involving chemically structural/compositional specificity to gut microbiota. Phytomedicine.

[25] Kim J., Han S.Y., Min H. (2018). Ginseng for an eye: Effects of ginseng on ocular diseases. J. Ginseng Res..

[26] Nishijo H., Uwano T., Zhong Y.-M., Ono T. (2004). Proof of the mysterious efficacy of ginseng: Basic and clinical trials: Effects of red ginseng on learning and memory deficits in an animal model of amnesia. J. Pharmacol. Sci..

[27] Lee M.J., Kim E.H., Rhee D.K. (2008). Effects of *Panax ginseng* on stress. J. Ginseng Res..

[28] Syed N.H., Kalyna M., Marquez Y., Barta A., Brown J.W. (2012). Alternative splicing in plants—Coming of age. Trends Plant Sci..

[29] Götz S., García-Gómez J.M., Javier T., Williams T.D., Nagaraj S.H., Nueda M.J., Robles M., Talon M., Dopazo J., Conesa A. (2008). High-throughput functional annotation and data mining with the Blast2GO suite. Nucleic Acids Res..

[30] Kumar S., Stecher G., Li M., Knyaz C., Tamura K. (2018). MEGA X: Molecular Evolutionary Genetics Analysis across Computing Platforms. Mol. Biol. Evol..

[31] Xu B., Yang Z. (2013). PAMLX: A graphical user interface for PAML. Mol. Biol. Evol..

[32] Crooks G.E., Hon G., Chandonia J.M., Brenner S.E. (2004). WebLogo: A sequence logo generator. Genome Res..

[33] Schneider T.D., Stephens R.M. (1990). Sequence logos: A new way to display consensus sequences. Nucleic Acids Res..

[34] Chen C.J., Chen H., He Y.H., Xia R. (2018). TBtools, a Toolkit for biologists integrating various biological data handling tools with a user-friendly interface. BioRxiv.

[35] Freeman T.C., Goldovsky L., Brosch M., van Dongen S., Mazière P., Grocock R.J., Freilich S., Thornton J., Enright A.J. (2007). Construction, visualisation and clustering of transcription networks from microarray expression data. PLoS Comput. Biol..

[36] Cardon G., Höhmann S., Klein J., Nettesheim K., Saedler H., Huijser P. (1999). Molecular characterisation of the Arabidopsis SBP-box genes. Gene.

[37] Guo A.Y., Zhu Q.H., Gu X., Ge S., Yang J., Luo J. (2008). Genome-wide identification and evolutionary analysis of the plant specific SBP-box transcription factor family. Gene.

[38] Wang J.W., Czech B., Weigel D. (2009). miR156-Regulated *SPL* transcription factors define an endogenous flowering pathway in *Arabidopsis thaliana*. Cell.

[39] Chuck G., Cigan A.M., Saeteurn K., Hake S. (2007). The heterochronic maize mutant Corngrass1 results from overexpression of a tandem microRNA. Nat. Genet..

[40] Wang J.W., Park M.Y., Wang L.J., Koo Y., Chen X.Y., Weigel D., Poethig R.S. (2011). miRNA control of vegetative phase change in trees. PLoS Genet..

[41] Franco-Zorrilla J.M., Valli A., Todesco M., Mateos I., Puga M.I., Somoza I.R., Leyva A., Weigel D., García J.A., Paz-Ares J. (2007). Target mimicry provides a new mechanism for regulation of microRNA activity. Nat. Genet..

[42] Todesco M., Rubio-Somoza I., Paz-Ares J., Weigel D. (2010). A collection of target mimics for comprehensive analysis of microRNA function in *Arabidopsis thaliana*. PLoS Genet..

